# Characterization of the Adsorption Mechanism of Manganese Phosphate Conversion Coating Derived Tribofilms

**DOI:** 10.1007/s11249-018-1082-2

**Published:** 2018-09-12

**Authors:** D. Ernens, G. Langedijk, P. Smit, M. B. de Rooij, H. R. Pasaribu, D. J. Schipper

**Affiliations:** 1 Laboratory for Surface Technology and Tribology, Department of Engineering Technology, University of Twente, P.O. Box 217, 7500 AE Enschede, The Netherlands; 2 Shell Global Solutions International BV, Innovation, Research & Development, Wells R & D, Kessler Park 1, 2288 GS Rijswijk, The Netherlands; 3 Engineering Physics, Faculty of Technology, Innovation & Society, The Hague University of Applied Sciences, Delft, The Netherlands

**Keywords:** Tribochemistry, Boundary lubrication chemistry, Phosphate conversion coatings, Oil production

## Abstract

Casing connections in the oil and gas industry are typically coated with zinc and/or manganese phosphate for corrosion protection during storage. The presence of phosphate coatings is also known to give beneficial tribological performance. The coating allows the system to run without problems long after it is worn off. This is because of two mechanisms. Glaze layer formation on the coated surface and, as will be shown, tribofilm formation on the uncoated counter-surface. An investigation into the mechanism behind this tribofilm formation is presented in this paper. The aim is to develop lubricants that exploit these mechanisms. A pin-on-disc set-up was used to investigate the interaction of a manganese phosphated disc and bare counter surface. Six base oils with different polarity and viscosity were used. The resulting tribofilms were analysed using optical microscopy, scanning electron microscopy, X-ray diffractometry, X-ray photoelectron spectroscopy, focused ion beam, and atomic force microscopy. The tribofilm is robust, amorphous, and only forms in the presence of a lubricant under sliding conditions and adsorbs on substrates with a large variation in chemical composition. It is concluded that the tribofilm consists of physisorbed manganese phosphate and formation is shear stress activated.

## Introduction

**Fig. 1:**
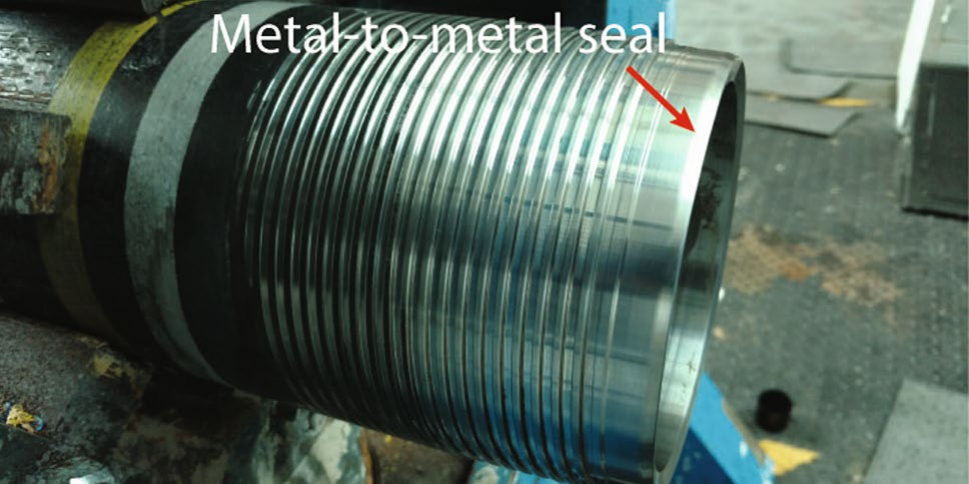
Male or “pin” member of a premium casing connection assembly. The pin is connected to a female or “box” member by the thread. At the final moment of assembly, the metal-to-metal seal engages ensuring a gas tight connection

Casing connections (Fig. 1) in the oil and gas industry are used to connect 12-m sections of pipe by means of a threaded male (pin) and female (box) member to fortify the bore hole during drilling [1]. The connections are (typically the box) coated with zinc and/or manganese phosphate for corrosion protection during storage. The presence of phosphate coatings is also known to give beneficial tribological performance [2–4].

The phosphate coating has mostly been ignored in the design of lubricants for the oil and gas industry. In the search for environmentally friendly lubricants, the presence of phosphate coatings should be exploited [3]. This can remove the high-temperature break down issues with currently available products as shown for instance in [5].

The protective properties on the coated side of the tribosystem have been shown to come from the formation of a hard and smooth glaze layer [2, 3, 6, 7]. However, this is not the full explanation. It was observed that on the uncoated counter surface a tribofilm was formed under unidirectional sliding conditions in [2, 3].

The authors are not aware of a study investigating the formation of tribofilms in phosphate conversion coated tribosystems. In all cases, the changes on the coated member are monitored [8] or of the system altogether for instance on the basis of the seizure resistance [9]. This is probably because it is mainly seen as a running-in coating [10] for long running systems. However, in a casing connection and the metal-to-metal seal, the sliding lengths during assembly are short ($<1$ m) and the sliding velocities are low ($<30$ mm/s). Given the low total sliding distance, the phosphate coating and its associated generated tribofilms affect the wear process strongly [3, 11]. The wear changes the surface topology of the metal-to-metal seal [12] and thus influences the subsequent seal ability [5, 13–15]. The mechanism behind the formation of this tribofilm, its properties, and the influence of the base oil on these properties with the aim of developing an environmentally friendly lubricant is the subject of this paper.

## Background

The phosphate coatings of interest consist of stable crystals of hopeite [16] (zinc phosphate, ZP) or hureaulite [17] (manganese phosphate, MP). The crystals have been formed under a conversion process involving an acidic solution with phosphate ions reacting with the steel substrate. Subsequently, the crystalline coating precipitates [6, 18]. The focus in this investigation is on manganese phosphate because of its more isotropic crystallites. However, similar tribofilm formation was observed in zinc phosphate [2].

The formation of tribofilms is important in contacts running in the boundary or mixed lubrication regime. Typically, the tribofilm is the last line of defence and if it fails the system transitions from mild wear to severe wear [19]. The film is formed by adsorption on and/or chemical reaction at the surfaces of the contacting members [20]. This can be initiated and accelerated by shear or thermal influences or a combination thereof [21]. According to a recent review, there is a wide range of additives employing one or a combination of these mechanisms to form the tribofilm [22]. Fatty acids, amides, and functionalised polymers for instance are physically adsorbed. On the other hand, stearic acid forms iron stearate with iron oxide by chemical adsorption. The simplest chemical reaction derived film is formed by sulphur which reacts with freshly exposed iron once the oxide layer is removed by wear [23]. In general, on steels, the generation of chemically reacted tribofilms is governed by temperature while this is typically not the case for ceramics [24]. Surface engineering on the other hand aims to modify the surface by applying a coating or make deliberate changes to the surface texture using for instance laser technology to functionalize the surface. Nano-composite coatings aim to do both: promote tribofilm growth and provide high hardness [25].

A well-known example of the interaction of phosphate chemistry with steel surfaces [26] and/or the properties of the base oil [27] is zinc dialkyldithiophosphate (ZDDP) which is widely used as anti-wear additive [28]. The growth rate is governed by the applied (shear) stress [29], base oil polarity [30], and bulk temperature which could be satisfactorily described by an Arrhenius growth model [29]. Still, the formation relies on contact between the sliding members and stops when the film thickness is significantly greater than the roughness i.e. the contact operates in the elastohydrodynamic lubrication (EHL) regime [31].

Following the approach of previous investigators, tribofilm growth because of rubbing against a manganese phosphate conversion coating is investigated in this paper. The working hypothesis was that tribofilm growth is governed by a chemical reaction and influenced by base oil polarity. To understand the mechanisms behind its growth, the thickness of the tribofilm is monitored as function of the normal load, sliding length, velocity, bulk temperature and base oil polarity.

## Experimental

The conditions during assembly of a casing connection have been studied in a contact situation where the pin is represented by a ball that slides against a phosphated flat representing the box [3]. Here only the assembly of the metal-to-metal seal is considered. During assembly, the pin and box are in continuous contact. The pin seal sees a stationary contact, whereas the box seal sees a moving contact because of the thread pitch. By subsequently selecting the right reciprocating amplitude, the continuous contact and the ratio of sliding lengths between the pin and the box can be properly simulated.

The reciprocating set-up has been chosen to easily simulate the assembly process for the study of the rate of formation and removal of the protective tribofilms on the uncoated ball surface under influence of sliding length, sliding velocity, contact stress, bulk temperature, base oil viscosity and base oil polarity. The combination of parameters will give insight in the particular adsorption or reaction mechanism responsible for the tribofilm formation. Next to that, the ball chemistry was varied to confirm the found adsorption mechanism of the tribofilm. For instance, if the mechanism is chemisorption or a chemical reaction on the bare steel surface no tribofilm formation is expected on stainless steels or ceramics [23].

### Lubricants

The influence of the base oil polarity is studied with commonly used base oils to select the base stock which maximizes the tribofilm formation. The relevant properties of the model fluids and base oils are listed in Table 1. The polarity was determined using the aniline point [32]. The dynamic viscosities were determined using the rheometer protocol listed in Sect. 3.3.

**Table 1:** Properties of the used commercial base oils ranked by polarity

Base oil name	Type	Polarity ranking	Aniline point ($^{\circ }{\text{C}}$)	Dynamic viscosity (22 $^{\circ }{\text{C}}$, 500 1/s) (Pa s)	Density (15 $^{\circ }{\text{C}}$) (${\text{kg/m}}^{3}$)
A	Group V	1	< 20.0^a^	0.166	915
B	PAG	2	30.0	0.383	1019
C	Group I	3	104.2	3.320	943
D	Group I	4	117.9	1.520	958
E	Group II	5	125.6	0.245	866
F	PAO	6	170.0	3.000	853

${^\mathrm{a}}$Taken from technical data sheet

### Materials

The experiments were performed using a 10-mm ball on flat configuration. The balls are polished and made from AISI52100 bearing steel, AISI316 stainless steel, aluminium oxide or silicon nitride. For the preparation of the flat, the same procedure as in [2] was used. The flat is made from quenched and tempered AISI4130 which was subsequently ground and then dip coated with manganese phosphate. The bare flat is made from the same material and polished. The properties of these materials are listed in Table 2. The choice for AISI4130 is because it has the same chemical composition as most oil field quenched and tempered casing material grades [3].

**Table 2:** Summary of used materials and their properties

Material name (–)	Surface finish Ra ($\upmu {\text{m}}$)	Young’s modulus (GPa)	Surface hardness (GPa)
AISI316L	0.01	160	2
AISI4130-1	0.01	210	3
AISI4130-2	0.10	210	3
AISI52100	0.01	213	7
Si3N4	0.03	300	35
Al2O3	0.03	360	20

### Rheometer

The base oil dynamic viscosities were measured using a Haake Mars III rheometer. The measurement geometry was bob (CC25) in cup (CCB25). Temperature was controlled using the Peltier module (TM-PE-C). The dynamic viscosity was measured by imposing a shear rate sweep of 0.01–500 1/s at a temperature of 22 $^{\circ }{\text{C}}$. In all cases, the fluids showed Newtonian behaviour. The results of those measurements are also listed in Table 1.

### Pin-on-Disc Tribometer

The formation of the protective tribofilm is investigated using a Bruker UMT-3 pin-on-disc tribometer. Loads were measured with the 6D load cell (TFH-50). Two drive modules were used for the experimental program. The low-velocity (0.05–0.5 mm/s) and-high temperature (200 $^{\circ }{\text{C}}$) reciprocating tests were performed with the slider of the tribometer combined with the high-temperature module (S35HE-350). The high-velocity ($>0.5$ mm/s) reciprocating tests were performed with a dedicated reciprocating drive module (RF33FE).

### Experimental Design

First, it was checked that the formation is indeed shear stress induced or if contact stress alone could initiate tribofilm growth by a 24-h static (no movement, constant load) test at a constant contact stress of 1 GPa. The static tests were performed with base oil E. Next, the mechanism of formation is studied under variation of sliding length, sliding velocity, contact stress, base oil viscosity and base oil polarity. The tribofilm is formed under reciprocating gross sliding conditions with a fixed stroke length of 0.5 mm. This gives a similar sliding length ratio as the pin-to-box sliding ratio during make-up of the metal-to-metal seal. The experimental design is summarized in Table 3. The sliding velocity is relatively low, and the related temperature increase under the testing conditions was computed to be negligible using [33]. Each data point is obtained with a fresh ball on a fresh patch of coating. Prior to the experiments, the ball was cleaned in acetone and heptane using an ultrasonic bath and subsequently wiped dry. The disc was only flushed with acetone and heptane to avoid removal of the coating by the sonication process and subsequently blown dry with nitrogen. After the experiment, the ball was flushed with acetone and heptane to remove the base oil and blown dry with nitrogen. From selected experiments, the ball with generated tribofilm was used on a bare polished disc to investigate the durability of the tribofilm.

**Table 3:** Overview of the range of experimental parameters used to study the formation of the tribofilm with a fixed stroke length of 0.5 mm

Normal force (N)	Cumulative sliding length (mm)	Sliding velocity (${\text{mm/s}}$)	Bulk temperature ($^{\circ }{\text{C}}$)	Base oil aniline point ($^{\circ }{\text{C}}$)	Base oil dynamic viscosity at 20 $^{\circ }{\text{C}}$ and 500 1/s (Pa s)	Maximum Hertzian contact stress (GPa)	Hertzian contact area ($10^{-3}\,{\text{mm}}^{2}$)
2.5–10	1–8000	0–10	22–200	20–170	0.245–3.3	0.47–1.0	3–15

### Analyses

The resulting tribofilms were investigated using a digital light microscope (Keyence VHX-5000), and measurements were taken with the accompanying software, X-ray Diffractometry (XRD, PANalytical Empyrean diffractometer, Cu anode, focusing optics: 0.1 and 0.3 mm monocapillary), Scanning Electron Microscopy (SEM, Tescan Vega 3) combined with Energy-dispersive X-ray spectroscopy (EDX), X-ray Photoelectron Spectroscopy (XPS, Kratos Axis Nova with 15 kV Al K$\alpha$ source), focused ion beam (FIB, FEI Helios NanoLab 650) and Atomic Force Microscopy (AFM, NanoSurf Flex-Axiom in tapping mode with ACTA cantilever). The analytical techniques were used to determine the following properties of the formed tribofilm: The area coverage, thickness, surface topography, (crystal) structure and composition as function of the experimental parameters (Table 3).

### Thickness Determination Based on Thin Film Interference

The maximum thickness of the tribofilms was determined by the colour scheme coming from the complex thin film interference using the optical microscope [34]. The method was calibrated with AFM and FIB/SEM measurements. Other methods including ellipsometry and interferometry were attempted; however, they either failed or were not precise enough because of the curvature and roughness of the ball or the complex thin film interference. The current method based on optical microscopy was determined to be accurate to ± 100 nm. Therefore, the method is only used to reveal trends.

### Data Processing

The measured data are post processed using Matlab ^®^ and the Curve Fitting Toolbox v2017a. The build in power law model power1 is used to fit

1 $$\begin{aligned} h(t) = at^b \end{aligned}$$

to the data where indicated, where *h* is the determined thickness in nanometers; *t* is the rubbing time in seconds, and *a*, *b* are the fitting parameters. To determine tribofilm growth rates, the resulting fit is differentiated with respect to time.

From the measurements of coefficient of friction (COF), $\mu$, normal force, $F_{\mathrm{n}}$, and tribofilm area coverage, $A_{\mathrm{cov}}$, an average shear stress can be computed according to

2 $$\begin{aligned} \tau = \frac{\mu F_{\mathrm{n}}}{A_{\mathrm{cov}}}. \end{aligned}$$

## Results

In the static tests, no tribofilm growth was observed. Next to that, the uncoated system is taken as a reference for tribofilm durability tests later in this section. For all base oils, the system failed within 0.10 m of sliding because of galling. When a base oil and sliding was included, tribofilm formation was subsequently consistently and repeatedly observed.

### Friction

**Fig. 2:**
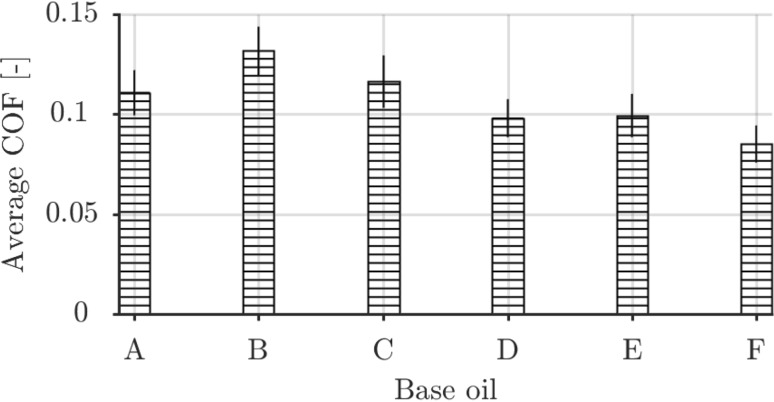
Average friction coefficients for all base oils. The error bars indicate 1 standard deviation for $N>$ 20 for each base oil

The average COF for the ambient temperature experiments is shown in Fig. 2. The average COF was determined in the growth region of the tribofilm formation in Fig. 4 to be able to compute meaningful shear stresses that (could) correlate with growth rate in Fig. 7. As shown, the COF was consistent for all base oils and sliding lengths with relatively small variance. The number of observations, *N*, per base oil differed, however, $N>$ 20 for each base oil.

### Tribofilm Formation as a Function of Sliding Length or Rubbing Time

**Fig. 3:**
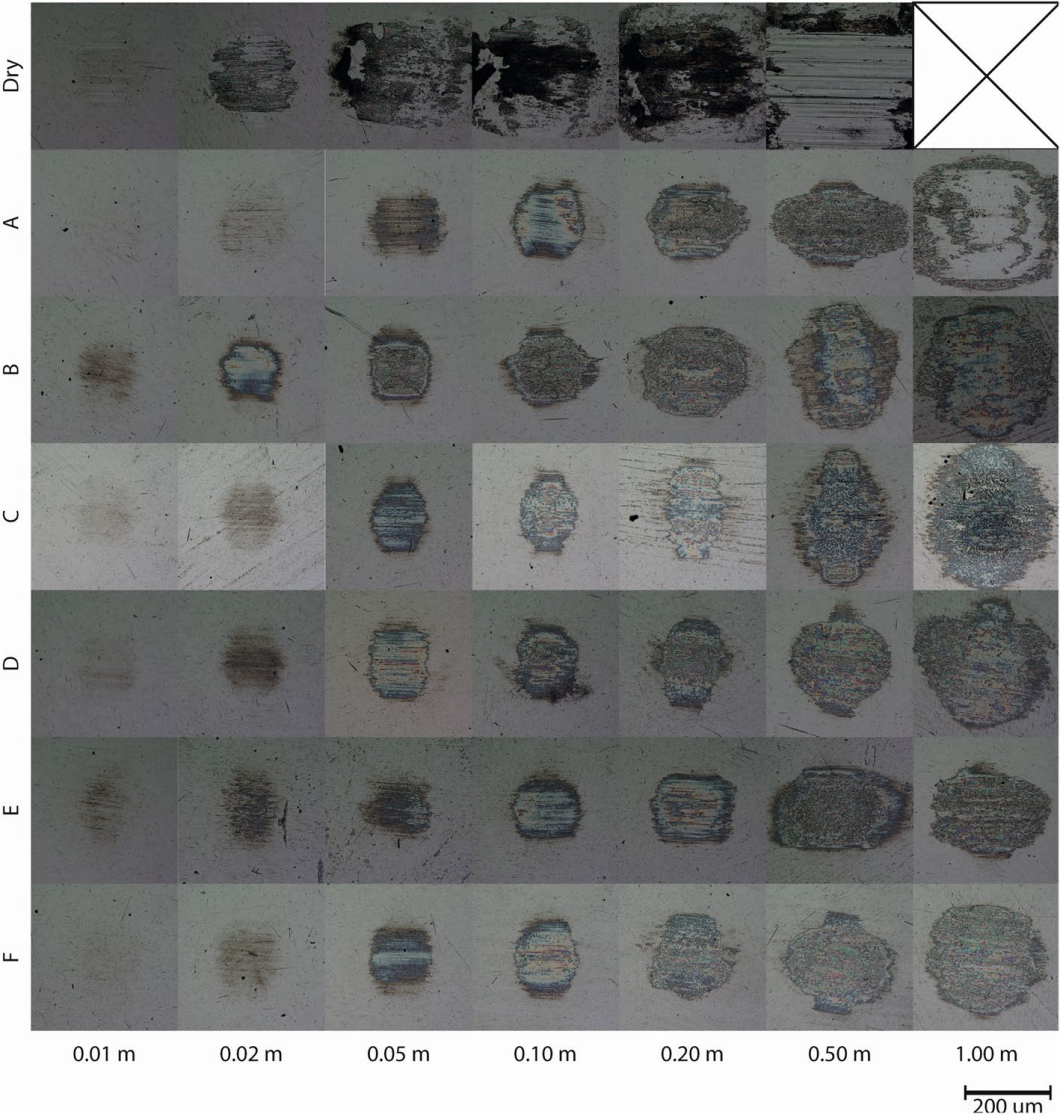
Overview of tribofilm formation as function of cumulative sliding length and base oil at a sliding velocity of 0.5 mm/s and temperature of 22 $^{\circ }{\text{C}}$. Reciprocating sliding direction is horizontal

The formation of the tribofilm was first studied as function of sliding length or rubbing time at a constant reciprocating velocity of 0.5 mm/s and 22 $^{\circ }{\text{C}}$. Figure 3 shows the light microscopy of the balls as function of the cumulative sliding length (columns) and base oil (rows). The reciprocating sliding is in horizontal direction. The top row shows the dry sliding experiments as a reference.

The following can be observed. In dry sliding conditions, no tribofilm is formed and complete hureaulite crystals are transferred. Furthermore, the system quickly wears through the phosphate coating and transitions to adhesive wear in line with previous investigators [11]. In lubricated conditions, a tribofilm readily forms for all base oils. The tribofilm has a patchy appearance. Based on the thin film interference, different tribofilm thickness and derived growth rates are observed for each base oil. In all cases, an increase in surface area coverage can be observed which could be indirectly used to compare the wear rates of the phosphate coating as function of the different lubricants.

**Fig. 4:**
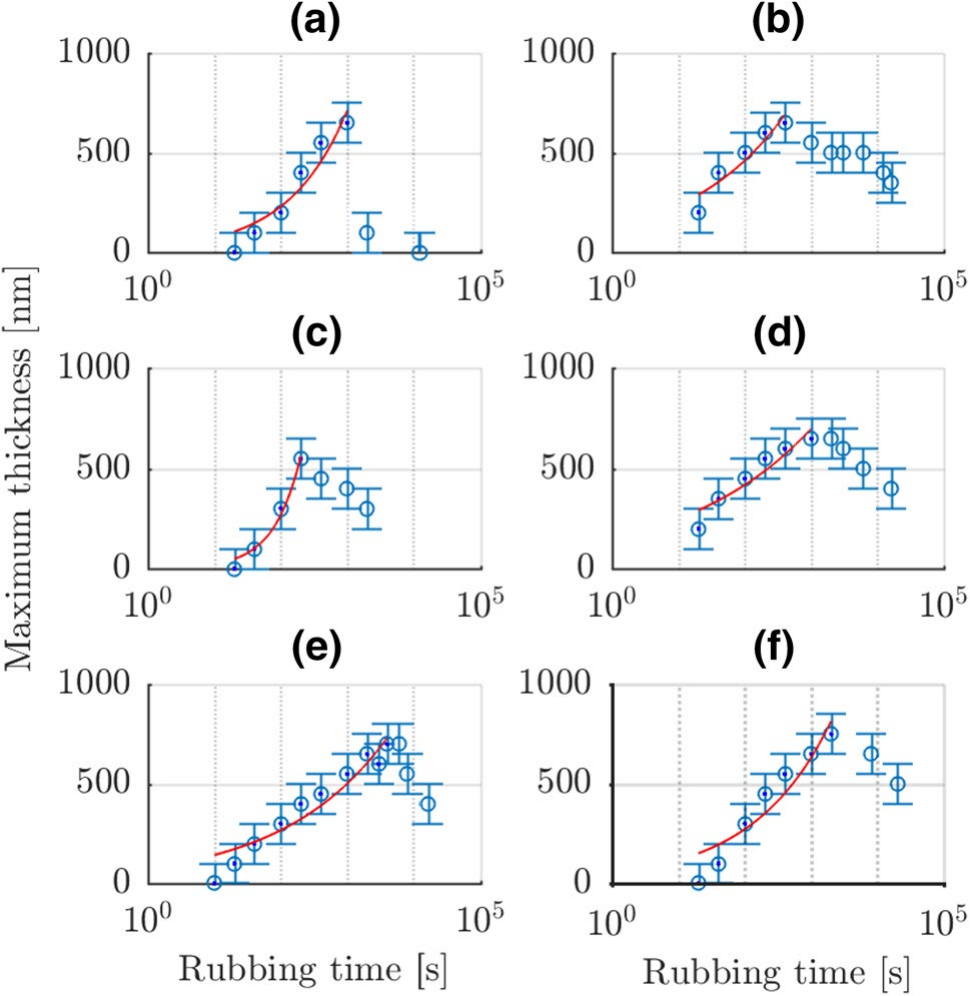
Tribofilm thickness evolution versus rubbing time at 0.5 mm/s and temperature of 22 $^{\circ }{\text{C}}$ with a power law fit in the growth region for all base oils. Closed points were used for the fit. The measurements up to 2000 s are corresponding with the images in Fig. 3

Using the results of Fig. 3, the thickness of the film can be determined based on the thin film interference (Sect. 3.7). The results of this are shown in Fig. 4 for each base oil. This gives the influence of the base oil on the layer thickness versus rubbing time and its power law fit (1). The fastest growth is observed for base oil B and the slowest for base oil F. All films grow to a similar limiting thickness before wear balances the growth. After that tribofilm wear reduces the thickness. Next to that, the counter surface phosphate coating also wears out. This reduces the supply of phosphate particles and therefore the growth rate up to the point that the ball is rubbing on bare steel (base oil A at 1 m in Fig. 3).

**Fig. 5:**
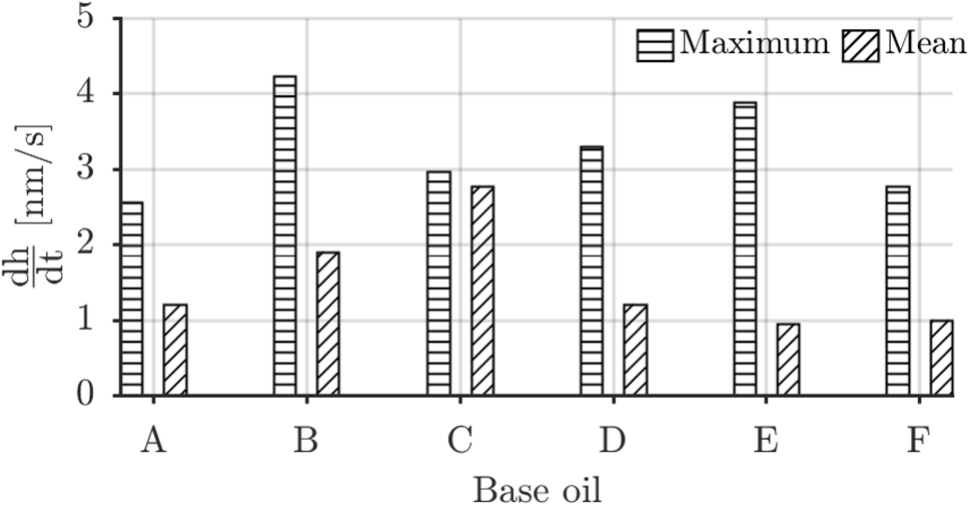
Maximum and mean tribofilm growth rate for all baseoils based on the derivative of the curve fits in Fig. 4

The growth rates for 10 N at 0.5 mm/s were determined by differentiating the fitting results of Fig. 4 against rubbing time and the maxima and mean values are shown in Fig. 5. The mean was determined in the region where $\dfrac{\text {d}h}{\text {d}t}>0$.

**Fig. 6:**
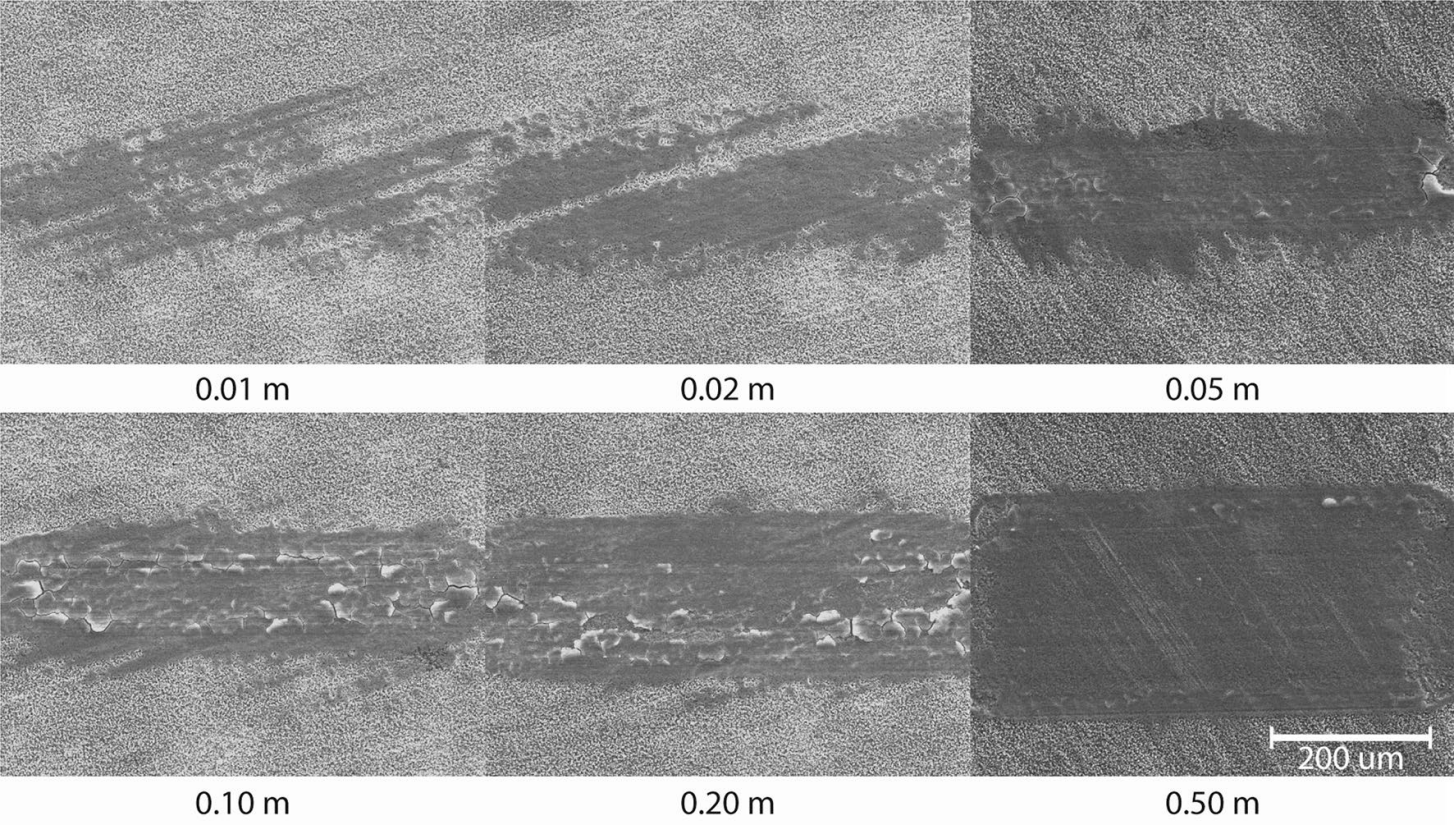
Secondary electron (20 kV) images of the wear scar for base oil B as function of cumulative sliding length at a sliding velocity of 0.5 mm/s and temperature of 22 $^{\circ }{\text{C}}$. The wear scars correspond to the tribofilms shown in Fig. 3

**Fig. 7:**
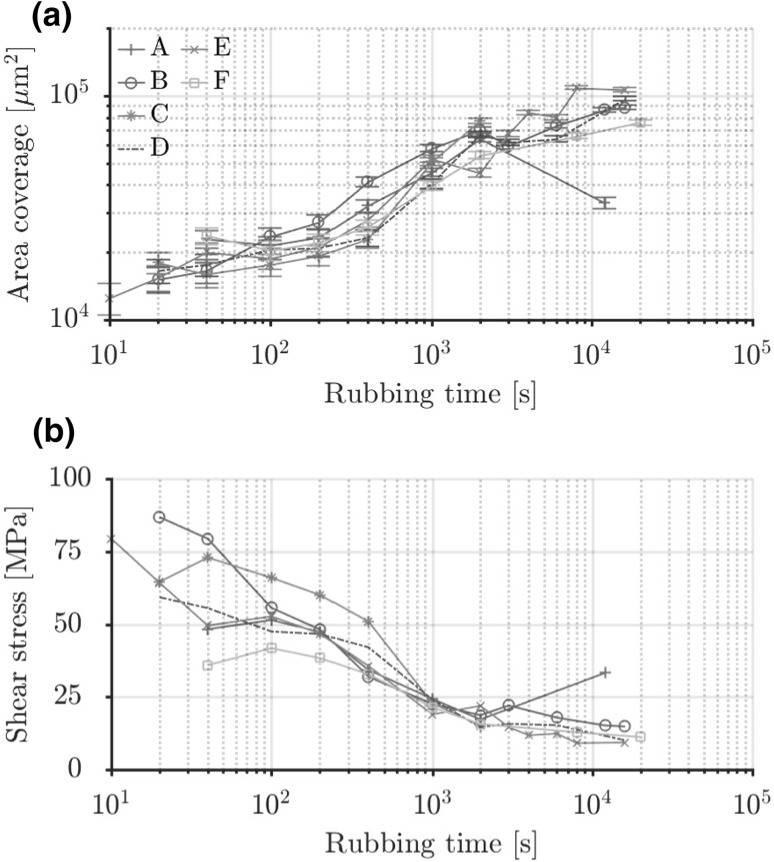
**a** Tribofilm surface coverage versus rubbing time. **b** Resulting calculated instantaneous shear stress as function of rubbing time

The measured tribofilm surface coverage as function of sliding length is shown in Fig. 7a. The increase of the area covered by the tribofilm is because of wear on the coated side of the tribosystem. This is shown for base oil B in Fig. 6 by the wear scar evolution as function of sliding length. The wear scar width corresponds to the tribofilm width for base oil B in Fig. 3. The ball sinks deeper in the coating increasing its area of contact. The increase in coverage is thus proportional to the wear of the coating on the disc. The measurements indicate that the initial wear is similar for all base oils and diverges after the maximum tribofilm thickness (Fig. 4) is reached. The divergence is because of removal of the tribofilm and coating at the heart of the contact which has seen most sliding length, see again for instance base oil A at 1 m in Fig. 3. This is compensated by tribofilm formation at the edges of the contact. The balance seems to be affected by the initial growth rate. The exclusive presence of the tribofilm in rubbed areas confirms the static test results and indicates a shear stress activation mechanism.

The shear stress activation hypothesis is tested by assuming that only rubbed area is covered by tribofilm. This allows computing of an average instantaneous shear stress using the measured friction and covered surface area data with Eq. (2). The result is shown in Fig. 7b as function of rubbing time. It is clear for instance that the high shear stress of base oil B and the low shear stress of base oil F correlate with their observed growth rates in Fig. 5. Next to that the shear stress is highest at the start of the rubbing experiment and reduces because the contact area grows as a consequence of the wear on the disc side. This matches the earlier assertion of wear balancing the growth; however, this is thus mainly because the shear stress goes down.

The tribofilm formation as function of force, temperature and velocity was further investigated with the oils presenting the highest (base oil B) and lowest (base oil F) growth rate hereafter.

### Tribofilm Formation as Function of Force, Temperature and Velocity

**Fig. 8:**
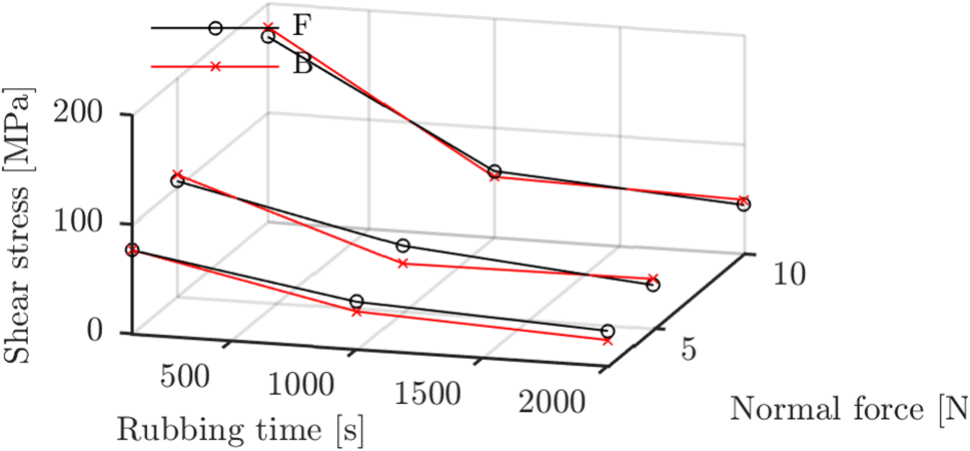
Calculated instantaneous shear stress as function of rubbing time and normal force at 0.5 mm/s and 22 $^{\circ }{\text{C}}$

**Fig. 9:**
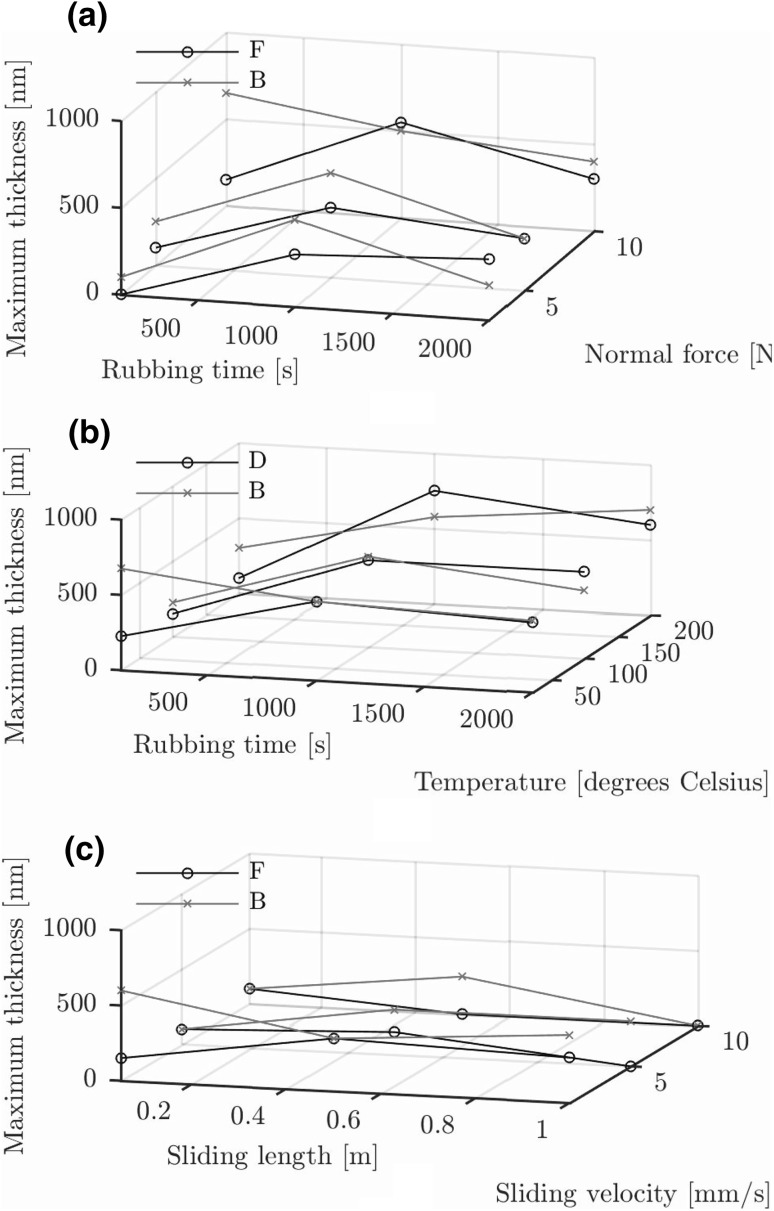
Tribofilm thickness as function of rubbing time and: **a** normal load at 0.5 mm/s and 22 $^{\circ }{\text{C}}$, **b** bulk temperature at 0.5 mm/s and **c** sliding velocity at 22 $^{\circ }{\text{C}}$. Note: Error bars of ± 100 nm removed for clarity of presentation

The stress activation mechanism and its influence on the tribofilm growth was investigated by varying the normal force. The results are shown in Fig. 9a. The thickness versus time measurements show a large influence in the first 100 s, particularly for base oil B. The influence is relatively small for base oil F. Converting the data to shear stress as shown in Fig. 8 indicates a similar result as Fig. 7b. The larger tribofilm thickness for base oil B is entirely because of the initially higher shear stress. After that, the shear stresses are comparable and the curves collapse on each other as shown in Fig. 8. The shear stress does increase with increased applied normal force explaining the increase in tribofilm thickness in Fig. 9a and further confirming the stress activation hypothesis.

The influence of bulk temperature on the tribofilm growth was investigated by increasing the bulk temperature of the disc and pin. The results are given in Fig. 9b. No acceleration of tribofilm formation by increasing temperature is observed for both base oils.

The results for increasing velocity are shown in Fig. 9c. The reduced tribofilm thickness for increasing velocities can entirely be attributed to increased wear at higher velocities of the tribofilm and the phosphate coating. In other words, it is not because of the shorter test duration giving less time for growth. Note further that this is not a thermal effect as discussed in Sect. 3.5.

### Tribofilm Topology and Thickness

**Fig. 10:**
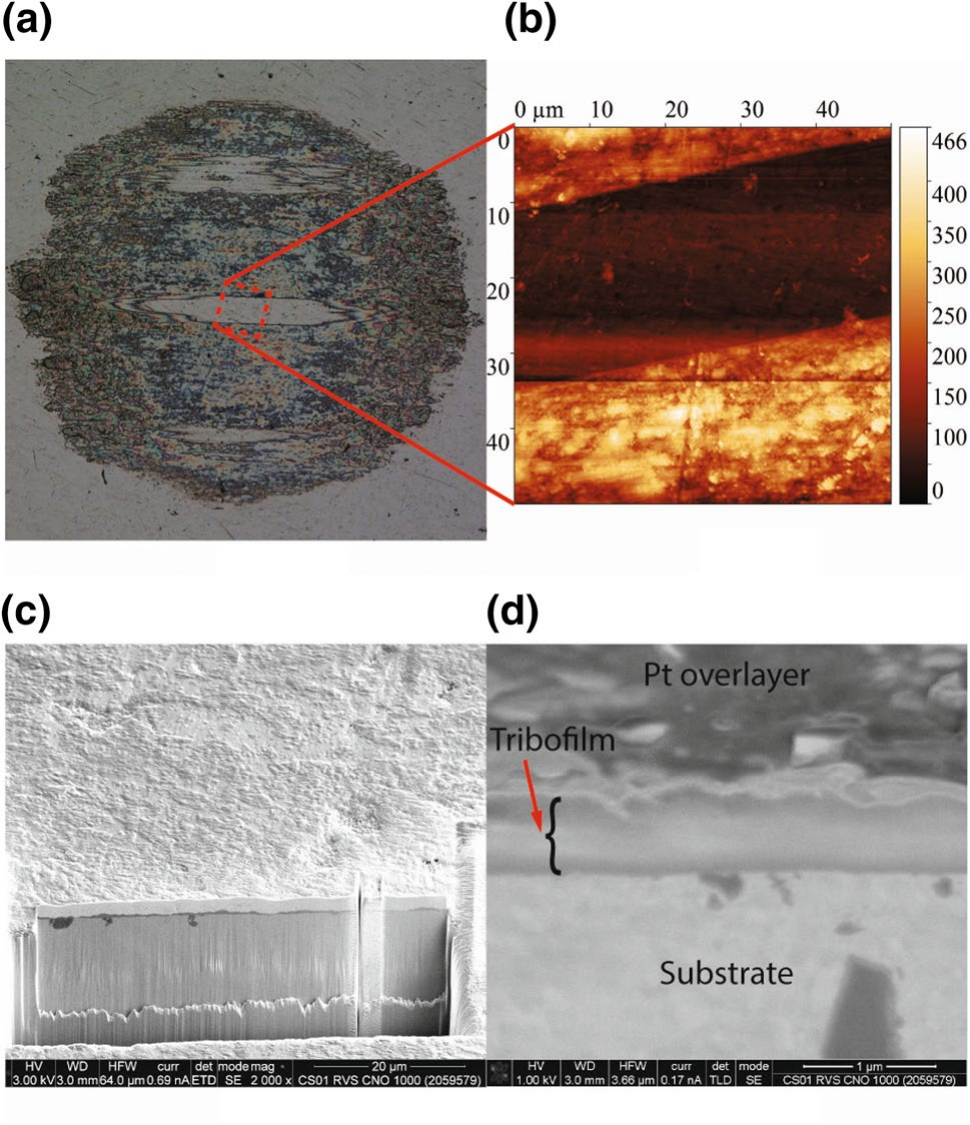
Surface topology and internal structure of the tribofilm. **a** and **b** show a LM image and AFM measurement of a tribofilm formed with base oil E after 8-meter sliding at 0.5 mm/s, 10 N and 22 $^{\circ }{\text{C}}$. *Note* approximate location is indicated. **c** and **d** show a SEM micrograph of the cross section made by FIB after 0.5-m equivalent sliding with base oil E at 0.5 mm/s, 10 N and 22 $^{\circ }{\text{C}}$

The surface topology of the tribofilm of a ball after 8-m equivalent sliding with base oil E at 0.5 mm/s, 10 N and 22 $^{\circ }{\text{C}}$ as shown in Fig. 10a was measured using AFM. This particular experiment was chosen because in the middle of the ball part of the tribofilm was removed showing the underlying ball surface. The measurement was performed at the edge of this opening in the tribofilm. The result is shown in Fig. 10b. The surface is still smooth indicating that roughening of the surface is not part of the tribofilm growth mechanism. It further confirms the earlier observations of a patchy tribofilm. After 8 m of sliding, the film had a maximum thickness of 466 nm. The surface was protected successfully either by the film itself or the separation that it created between disc and ball.

In addition, a cross section was made by FIB on another ball after 0.5-m equivalent sliding with base oil E at 0.5 mm/s, 10 N and 22 $^{\circ }{\text{C}}$. The cross section was investigated with SEM and shown in Fig. 10c and a zoom in of the tribofilm in Fig. 10d. The thickness is about 400 nm. No particular structure can be discerned in the tribofilm indicating a homogeneous composition throughout the thickness.

### Tribofilm Composition

**Fig. 11:**
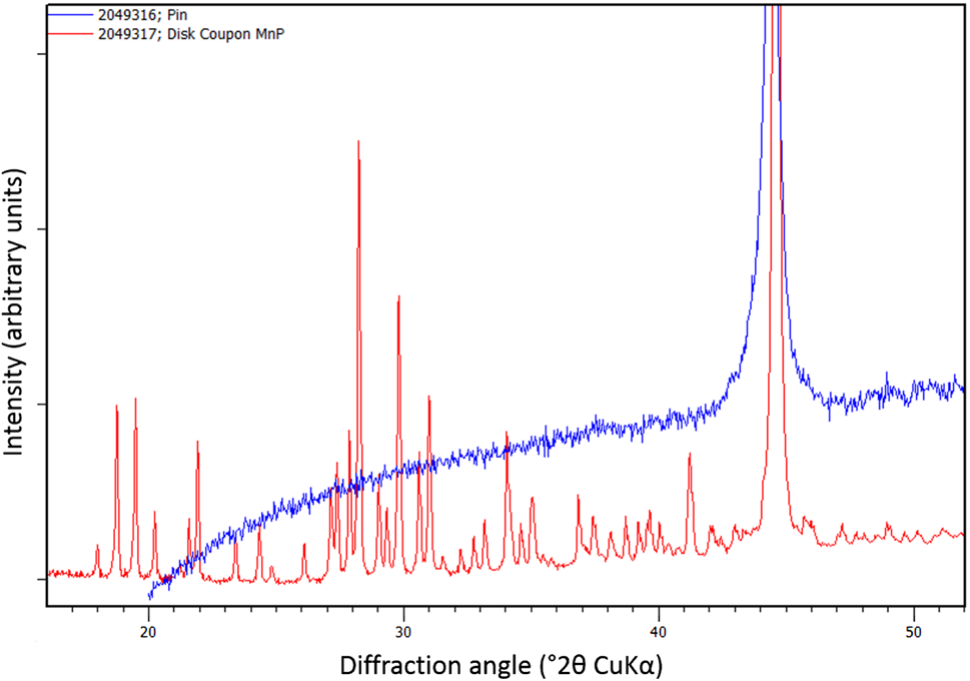
X-ray diffraction measurements of as received manganese phosphate coating compared with the tribofilm on the ball

The X-ray diffractogram is shown in Fig. 11. The untouched coating on the disc was taken as a reference to compare to the tribofilm on the ball. The disc diffractogram shows the characteristic Bragg reflections for hureaulite [17]. The reflections are sharp, corresponding to an avarage crystalite size of more than 100 nm (according to Scherrer’s equation, [35]). The tribofilm shows no Bragg reflections except for a strong Fe peak at $44.6^{\circ }$ coming from the underlying AISI52100 steel. If the hureaulite would have been broken up into crystal fragments of a few nanometer coherent length (i.e. approximately 5–10 unit cells), there still should be recognizable reflections in the diffraction pattern. This indicates that there are no crystalline phases present in the tribofilm which can therefore be considered to have an X-ray amorphous structure.

To validate the measurement method used for the tribofilm on top of the ball, a test sample was made by lightly dusting the top of a clean reference ball with rutile (${\text{TiO}}_{2}$) powder. Data collection using optical elements to selectively irradiate an area of approximately 0.3 mm radius resulted in a diffraction pattern where the applied titania phase could be readily identified.

**Fig. 12:**
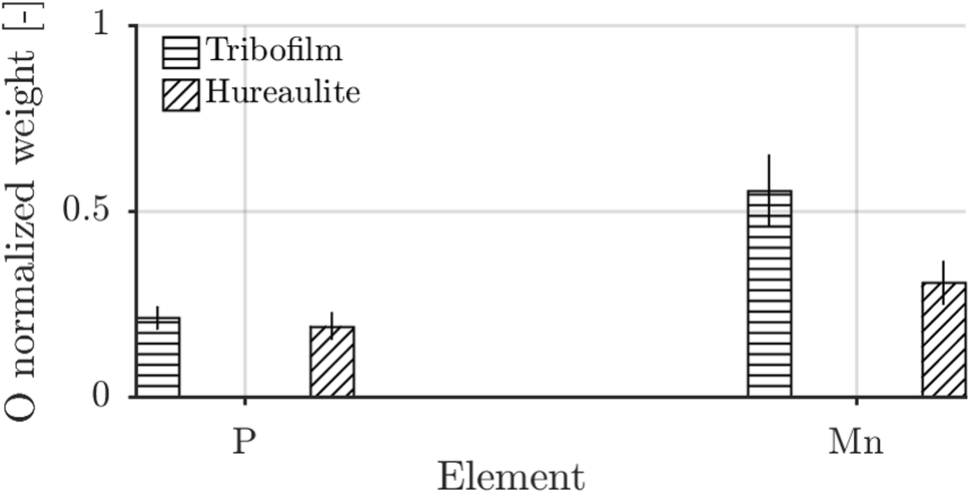
EDX measurement normalized by the respective oxygen weight content for the tribofilm on the ball and hureaulite on the disc surface

SEM-EDX shows (Fig. 12) that the tribofilm comprises of phosphorous, oxygen and manganese. The result is normalized with the oxygen content. Multiple ball surfaces were probed in regions with a thin film ($<100$ nm) and a thick film ($>400$ nm) generated with base oil B, base oil D and base oil F. It was observed that the absolute concentrations depended on the thickness but that the ratios were independent of thickness. No differences were observed between the base oils. The tribofilm P/O ratios matched the as received P/O ratio. This probably indicates that the tribofilm is still containing phosphate. A difference can be observed, however, for the manganese weight content (${\text{Mn}}_{\mathrm{film}}/{\text{Mn}}_{\mathrm{coat}}$ = 1.8) that could not be solely explained by the added manganese content of the underlying AISI52100. Even though this does not say anything about the molecular structure of the tribofilm and EDX is semi-quantitative it was interesting to note the ratio difference in the light of the possibility of a chemisorption or chemical reaction.

**Fig. 13:**
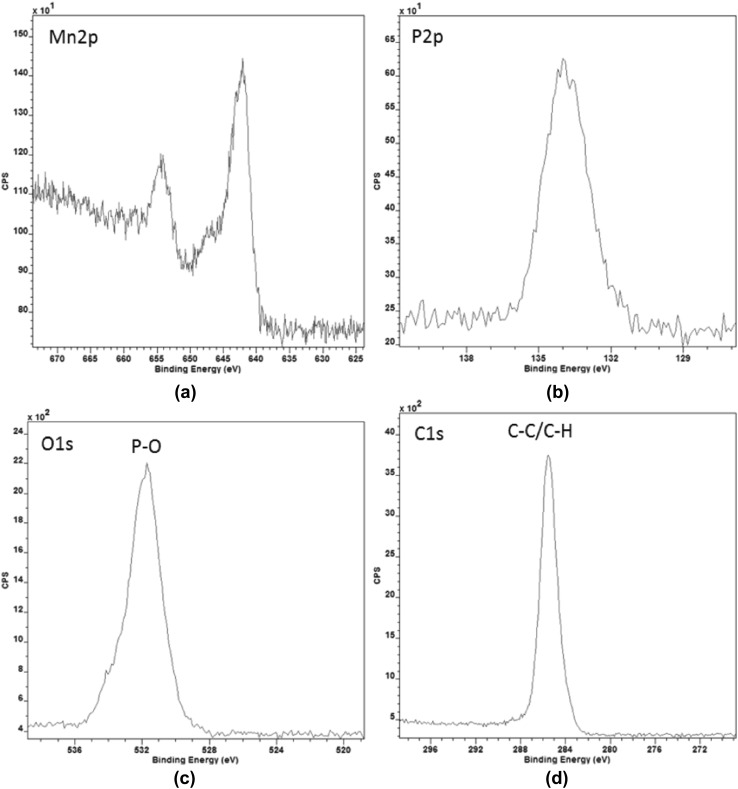
XPS measurement using a line scan of the first monolayers of a tribofilm generated with base oil E on a AISI316L ball

The assertions of SEM-EDX were confirmed and made more precise using XPS. A line profile was taken across the centre line of the film including the bare AISI316L steel surface on each side. The spectrum is measured on the tribofilm. Therefore, mainly the elements Mn, P, O, C are measured and to a lesser extent the elements Fe, Cr, N and Si. The spectra of the Mn2p, P2p, O1s and C1s peaks are given in Fig. 13. The binding energy and the shape of the Mn2p peak indicate a 2+ oxidation state of Mn (Fig. 13a). The P2p peak indicates the presence of one P-containing component. The binding energy of the peak can be associated with a phosphate phase (Fig. 13b). The O1s peak indicates the presence of more than one O-containing component. The main peak at a binding energy of 531.7 eV could be associated with the phosphate phase (Fig. 13c). The C1s peak indicates the presence of only one component (Fig. 13d).

**Fig. 14:**
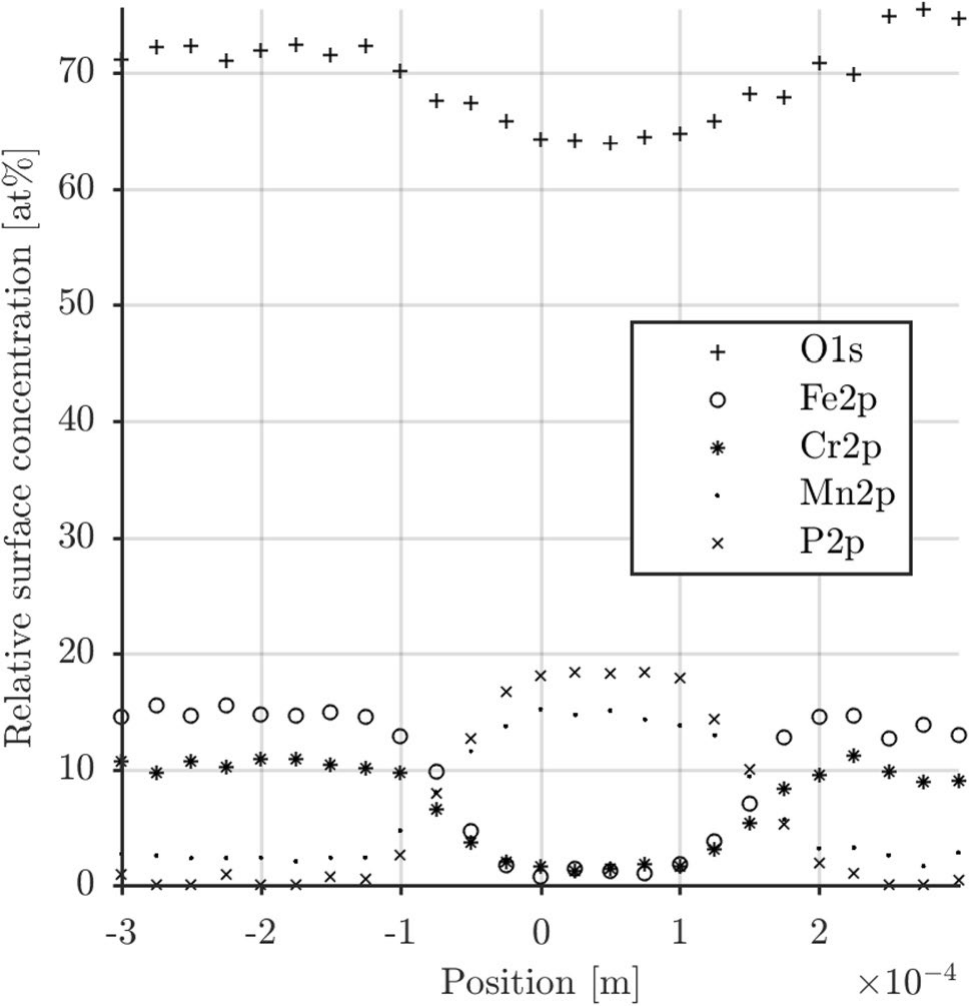
XPS linescan result belonging to Fig. 13

The line profile revealed a high C surface concentration. This probably comes from adsorbed base oil or solvent. For comparison of concentrations, the composition was calculated leaving the c signal out. The result is the line profile in Fig. 14. The line profile reveals a Mn/P ratio of  0.8. If a simple correction [36] is made for the carbon-overlayer and the Mn/P ratio becomes  1.3. Which is a similar ratio between these elements as the EDX (Fig. 12) of hureaulite and it is now clear that the tribofilm consists of a phosphate. It was concluded based on the Mn/P ratio, the 2+ oxidation state and knowledge of the source of the tribofilm that this is probably hureaulite with the molecular formula of ${\text {Mn}}^{2+}_{5} ({\text {PO}}_{3}{\text {OH}})_{3}({\text {PO}}_{4})_{2} \cdot 4 {\text {H}}_{2}{{\text {O}}}$ [17]. Another candidate could be ${\text {Mn}}_{3}({\text {PO}}_{4})_{2}$, which is the simplest phosphate molecule fitting the findings.

The observations with the initial line profile were confirmed by sputtering the tribofilm for a certain amount of time after which the composition is measured. Sputtering was done using 5 kV Argon ions. The sputtered area was 1 $\times$ 1 mm, hence covering the entire tribofilm and part of the untouched ball surface.

**Fig. 15:**
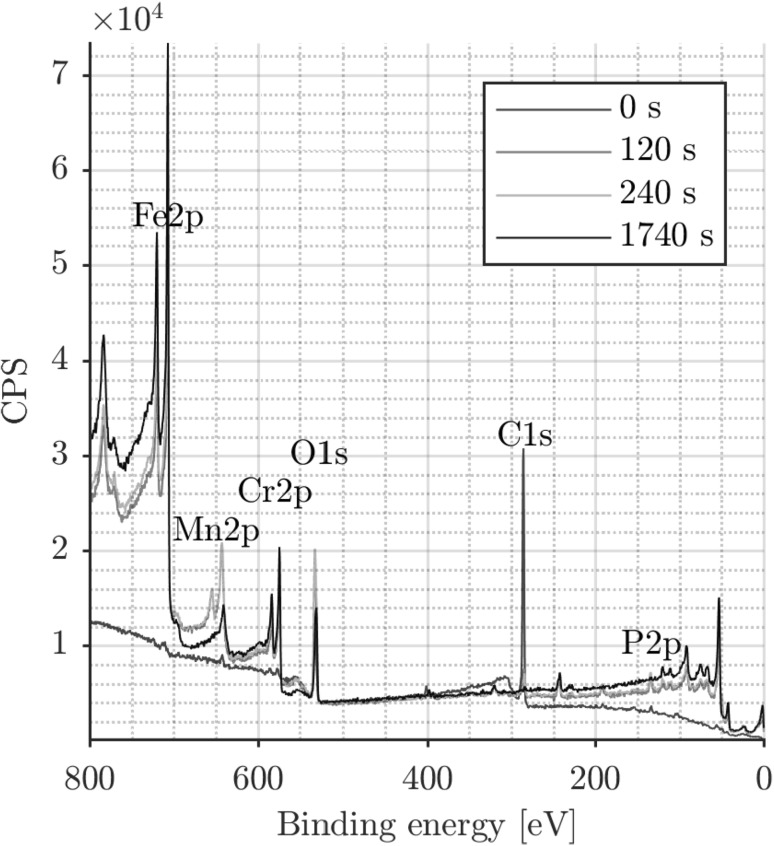
XPS depth spectra after sputtering

The tribofilm was sputtered in three steps. The resulting spectra are shown in Fig. 15. The initial spectrum (0 s) shows the earlier discussed overwhelming C1s peak. After 120 s of sputtering, the C1s peak has decreased in intensity considerably. The sputtering has removed the carbon containing top layer revealing the layer underneath. The binding energies of Fe2p and Cr2p in the initial spectrum (0 s) indicated that most of the Fe and Cr is present in an oxidized state. After sputtering, only the metallic state of both elements is observed. The spectrum after 240 s is similar to the 120 s spectrum. The Mn2p and P2p peaks decreased in intensity and the Fe2p and Cr2p peaks increased in intensity. After 1740 s, the Mn2p and P2p peaks disappeared from the spectrum indicating complete removal of the tribofilm. This was confirmed by optical microscopy. This rapid removal was surprising and indicated a 4–8 times higher sputter yield than anticipated.

Comparing the 0, 120 and 240 s results and using the knowledge of the higher sputter yield, and thus larger steps in depth, it is clear there are no compositional differences throughout the thickness. This is in line with the FIB/SEM observations in Fig. 10d.

### Tribofilm Durability

**Fig. 16:**
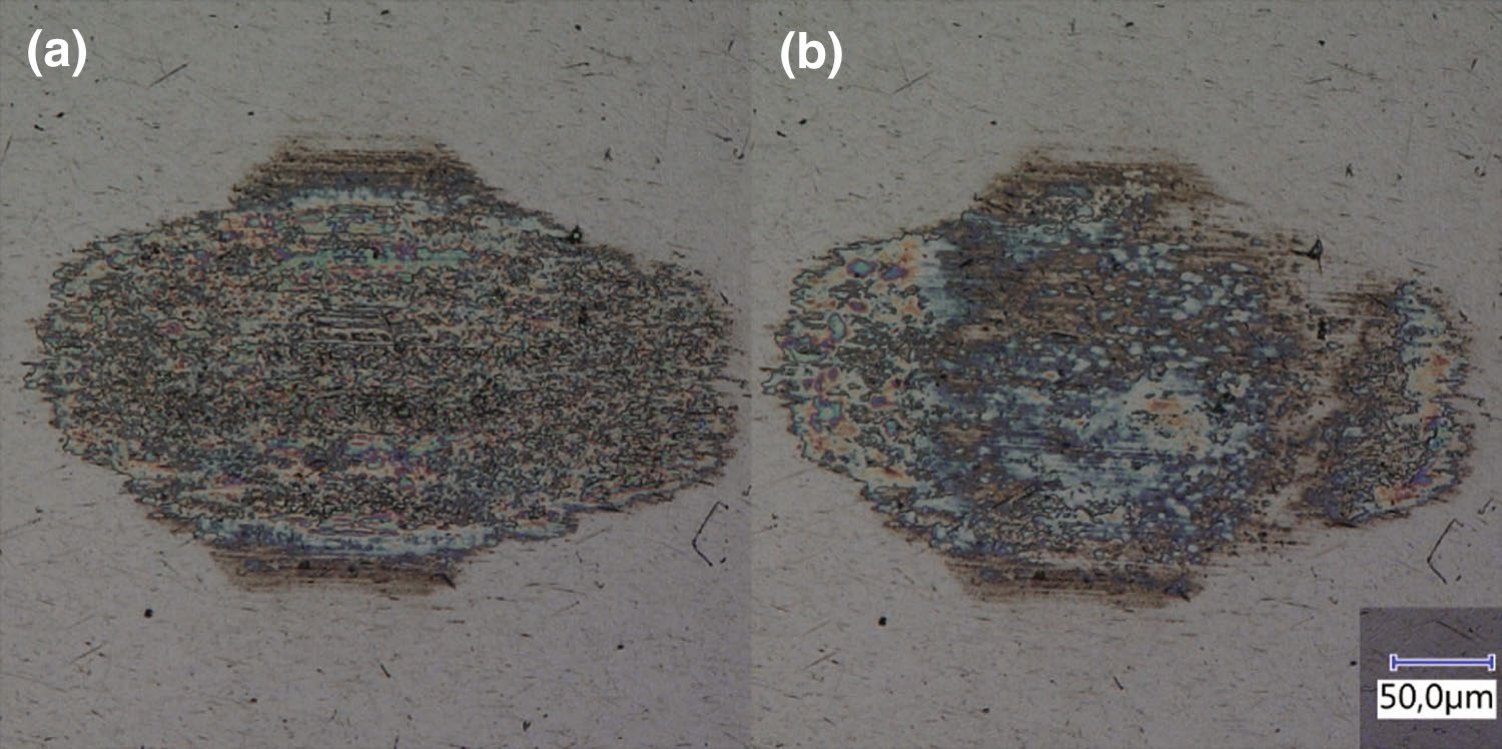
Tribofilm growth **a** on the phosphated disc after 0.5-m sliding at 0.5 mm/s, 10 N and 22 $^{\circ }{\text{C}}$. Removal **b** on the polished disc for base oil A after 0.05-m sliding at 0.5 mm/s, 10 N and 22 $^{\circ }{\text{C}}$

The durability of the tribofilm was investigated by generating a tribofilm with a lubricant. The tribofilm was generated over 0.5 m equivalent sliding at 0.5 mm/s, 10 N and 22 $^{\circ }{\text{C}}$. The disc was subsequently exchanged for an uncoated and polished disc to test the durability. An example is shown for a tribofilm generated with base oil A. The test on the uncoated disc was run for 0.05 m with the same parameters and lubricant. The result is shown by the comparison between before and after in Fig. 16. The tribofilm has seen limited wear and shows that once the film is formed it is protecting the tribosystem successfully. This was repeated for all base oils and longer sliding lengths without tribosystem failure.

### Tribofilm Growth on Stainless Steel and Non-metallic Surfaces

**Fig. 17:**
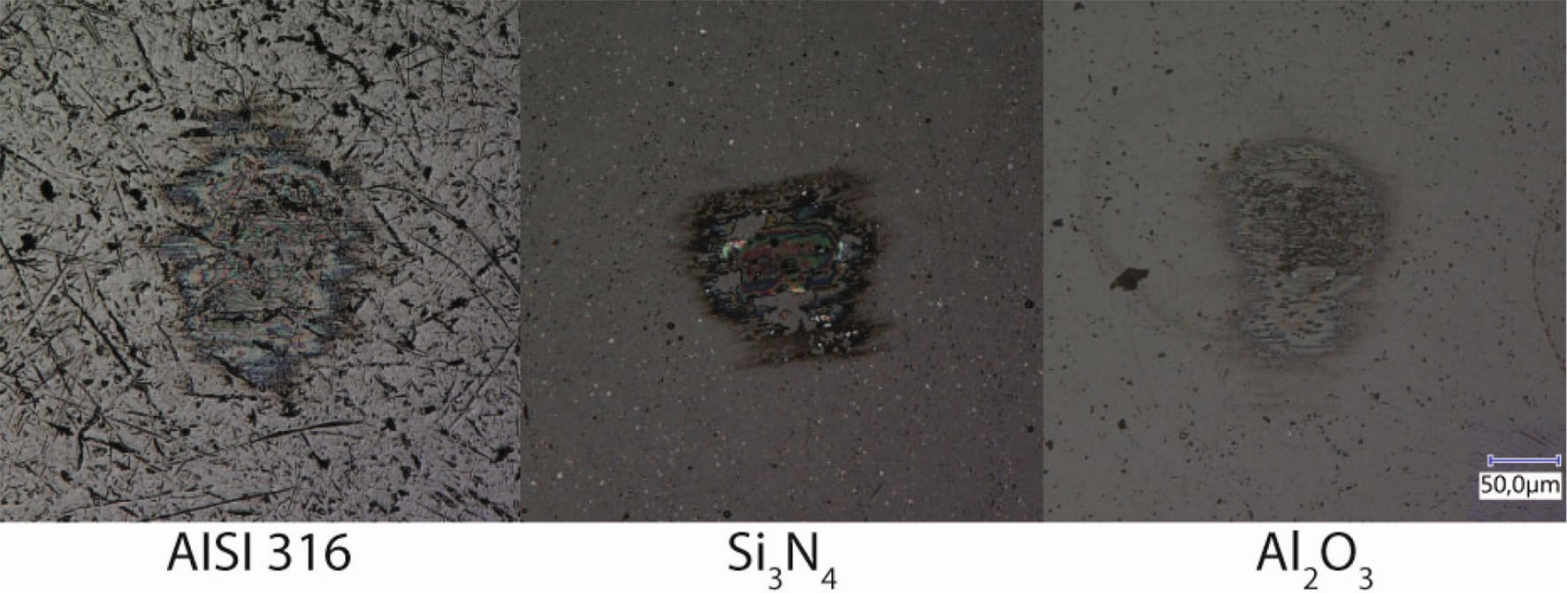
Tribofilm growth after 50 cycles with base oil B for different surface chemistries

The growth of the tribofilm was not unique for carbon steel surfaces. Meaning that the presence of (an) iron (oxide) is not a necessary condition for tribofilm growth. The results after rubbing for 50 cycles with base oil B for a selection of materials are shown in Fig. 17. It is observed that it forms readily on stainless steel (AISI 316) or non-metallic surfaces (Si$_3$N$_4$/Al$_2$O$_3$) surfaces. However, the growth rates were lower compared to carbon steel.

### Tribofilm Growth with Phosphate Rich Oil

Based on the previous results, it is interesting to investigate if tribofilms grow on polished uncoated steel discussing the harvested base oil with phosphate particles. If this is the case, these particles could also be considered as additives by adding them to the oil and remove the need for phosphating altogether.

**Fig. 18:**
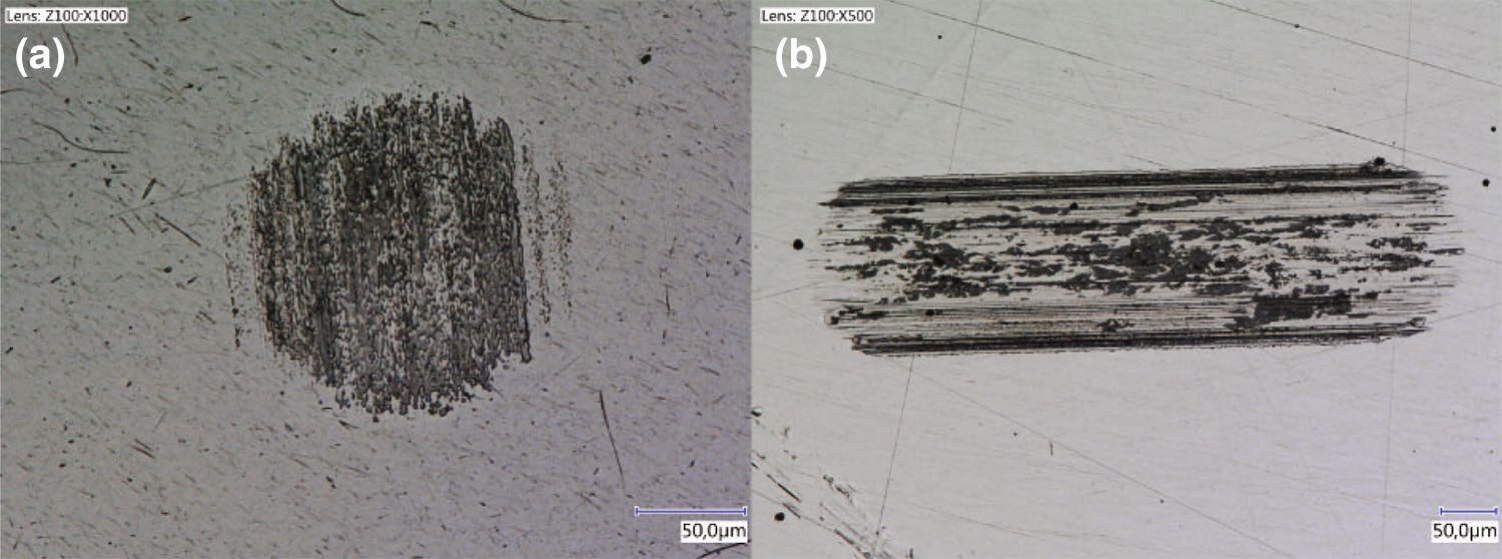
Pin and disc wear mark after sliding with harvested phosphate particle-rich oil

To obtain base oil with dispersed particles, the phosphated discs were washed after each experiment with ethanol. The solution of ethanol, base oil and particles was collected in a beaker. Next, the beaker was placed in an oven at 90 $^{\circ }{\text{C}}$ to evaporate the ethanol. The phosphate-rich oil was subsequently used to lubricate a polished disc versus polished ball contact using the same experimental settings as before. The results were rather disappointing, no tribofilm formation was observed with any of the base oils. Figure 18 shows the result after sliding 50 cycles. Signs of adhesive wear and scuffing can be clearly seen on disc and pin indicating that no protective film was formed. In addition, the same results were obtained with bought manganese phosphate powder (Sigma Alderich).

**Fig. 19:**
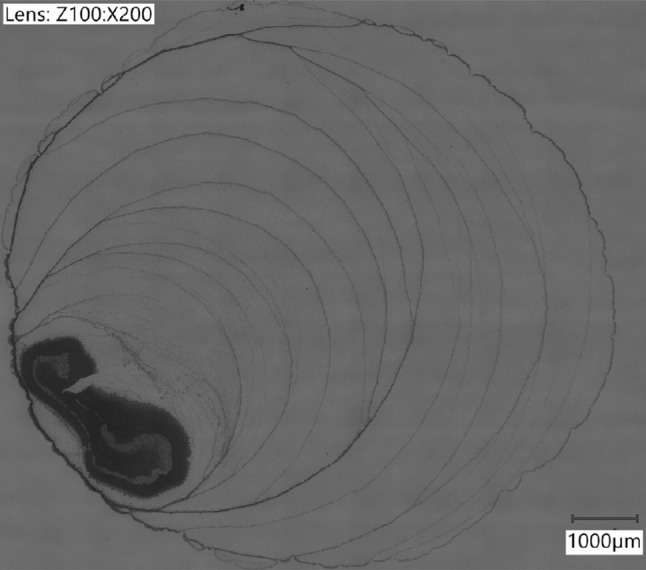
Results after evaporating the base oil from harvested oil from the rubbing experiment

To confirm that particles are dispersed, a set of samples were left in the oven until all the base oil was evaporated. The result is shown in Fig. 19. It clearly shows that debris particles were dispersed in the oil and are most probably responsible for the tribofilm growth.

### The Role of the Phosphate Coating

**Fig. 20:**
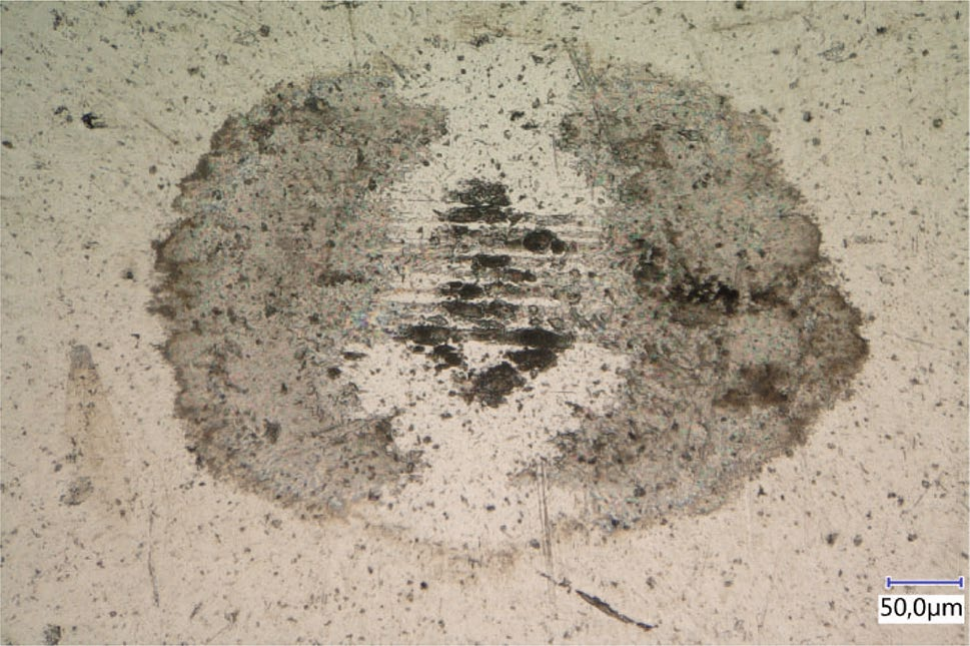
Tribofilm (or lack thereof) after sliding with base oil C on a thicker manganese phosphate coating after 0.5-m sliding at 0.5 mm/s, 10 N and 22 $^{\circ }{\text{C}}$

The thickness of the phosphate coating on the counter surface turned out to be an important factor in successful tribofilm growth. Tribofilms would only grow on the ball with coatings that were $<{3}\,{\text {g}}\,{\text {cm}}^{-2}$. On thicker coatings, large chunks of coating were removed because of ploughing and delamination [2]. This was because a thicker coating bears the subsurface stresses [37]. A ball surface after sliding on a thicker coating is shown in Fig. 20 for an AISI316 ball and base oil C. Hence for the mechanisms described in the previous chapters to occur the phosphate coating needs to act as a shear layer.

## Discussion

The tests results shown here support the hypothesis that tribofilm growth in a lubricated bare against phosphate conversion coated contact is governed by the applied shear stress in two ways. First, it breaks off small particles from the phosphate coating which get dispersed in the base oil (that is why the static tests did not yield a film). Second, it stress activates the particle such that it adsorps to the ball. The lubricant polarity was proven to not play a significant role in the tribofilm growth. However, the growth rate is inversely affected by velocity as shown in Fig. 9c. This was attributed to the increased wear of the tribofilm and the coating on the disc.

It is known that for proper dispersion of particles in a solvent, the particle polarity should be matched with the polarity of the solvent. Next to that, polar particles are dispersed by polar solvents and vice versa and incompatibility of particle and solvent leads to flocculation or agglomeration [38]. The role of the base oil polarity could be that, when it is properly matched (base oil B), it promotes dispersion of the generated particles and counters agglomeration. This maximizes the (re)activity and availability of the particles. On the other hand in an apolar solvent (base oil F), the particles tend to agglomerate which reduces their (re)activity and availability until a certain saturation threshold is reached.

The film formation is observed to only happen in the presence of a lubricant, the onset to be most rapid for (low viscosity) polar base oils and follows a power law as function of sliding length. Once the film is formed, the thickness and growth rates converge to similar values indicating that once the surface is covered and the concentration of dispersed manganese phosphate particles is high enough the base oil is not influencing the growth any more. This is confirmed in the subsequent tests with a variation in load, velocity or temperature. In all cases, the film thickness stopped increasing at 600–700 nm. This is probably because an equilibrium is reached between growth and removal similar to observations in ZDDP-derived films by [39]. The question is: can the formation be fully attributed to high friction (shear) and is this because of the low viscosity or the polarity?

**Fig. 21:**
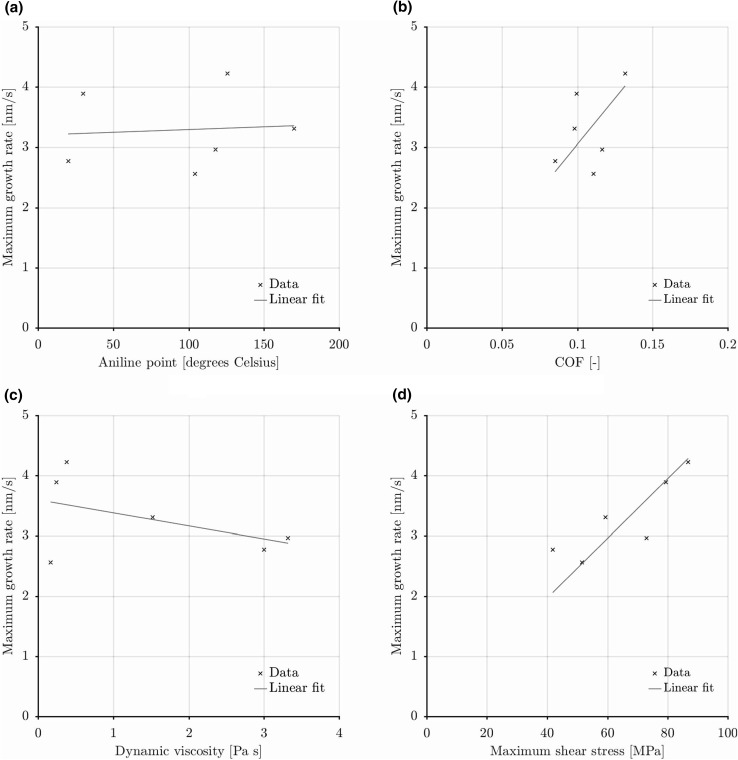
Correlation of maximum growth rate with the aniline point, COF, dynamic viscosity and maximum shear stress

The found maximum rates (Fig. 5) are plotted against base oil aniline point, COF, dynamic viscosity and maximum shear stress in Fig. 21. Starting with aniline point (Fig. 21a) no correlation was found in the data. Observational evidence (Fig. 3) did show early initiation for base oil B which might be attributed to its molecular structure instead of its polarity when taking into account the performance of base oil A (poly alkylene glycol vs adipate ester). There is a clear correlation with COF in Fig. 21b. This can weakly be attributed to the difference in base oil dynamic viscosity (Fig. 21c). The main effect though is because of the stress activation mechanism, the shear stress (and thus the COF) gives a clear correlation with the maximum growth rate as shown in Fig. 21d.

The tests with varying load, temperature and velocity (Fig. 9) confirm this and show that growth is directly proportional to shear stress and therefore indicates a stress activation mechanism. No acceleration was found when the bulk temperature was increased which rules out a chemisorption or chemical reaction mechanism. Therefore, the tribofilm formation is probably driven by physisorption.

The gradient of Fig. 21d can be used to estimate the interaction volume using the stress-promoted thermal activation equation following the work by [40] to speculate about the reactive group driving the physisorption. The equation is written as

3 $$\begin{aligned} {\text {Rate}} = A \exp \left( \frac{-\left( E - N \tau \Delta v \right) }{RT} \right) \end{aligned}$$

where *A* is the pre-constant, *E* the molar thermal activation energy, *N* Avogadro’s constant, $\tau$ the shear stress, $\Delta v$ the activation volume, *R* the gas constant, and *T* is the temperature. As no thermal activation was found, equation (3) can be simplified to

4 $$\begin{aligned} {\text {Rate}} = A \exp \left( \frac{N \tau \Delta v}{RT} \right) . \end{aligned}$$

Now the build up rate of the hureaulite tribofilm should be related to shear stress by taking the $\log$ of Eq. (4) such that

5 $$\begin{aligned} \log ({\text {Rate}}) = \log (A) + \frac{N \Delta v}{RT} \tau . \end{aligned}$$

The activation volume is found from the slope of the shear stress against a log(maximum growth rate) plot and calculated to be $0.052\,{\text {nm}}^{3}$. If a typical bond length of $0.2\,{\text {nm}}$ is considered, the molecular area over which the shear stress acts is $0.26{\text {nm}}^{2}$. The most plausible functional groups in the hureaulite molecule are the phosphate group, ${\text{PO}}^{3-}_{4}$, and the hydroxide group, ${\text {OH}}^{-}$. Using the bond lengths reported in [17] and the Van der Waals radii of the atoms, these groups yield a molecular area of roughly $0.29{\text { nm}}^{2}$ and $0.23{\text { nm}}^{2}$ respectively. In addition, the activation can be from deforming or stretching a single P–O bond in the phosphate group giving an area of $0.07{\text { nm}}^{2}$. As discussed, for adsorption to happen, particles need to be generated first. A crystal has a tendency to split along crystallographic structural planes because of relative weakness in the crystal which is known as cleavage. In other words, this can be seen as a preferential fracture plane. For hureaulite, this is perpendicular to the a-axis [17] in the unit cell abc-reference system and also denoted as {100}-plane [41] which will expose phosphate groups. Hence, the most plausible explanation for the adsorption of hureaulite is deformation or stretching of (a bond in) the exposed phosphate group. This will make the non-polar phosphate group slightly more polar leading to adsorption on the ball surface.

The experiments with harvested base oil or adding manganese phosphate powder to the base oil (Sect. 4.8) confirm this (re)activity and shows that this is lost if the oil with particles is isolated and reused on another disc. This is probably because the particles are quickly oxidized or hydrolised and explains why the tribofilm is only formed in situ. This is also why the manganese phosphate powder does not work as stabilizing agents are added during grinding to avoid agglomeration.

These assertions are further fortified by the patchy (Fig. 10) and amorphous (Figs. 10d, 11) structure, the unchanged chemical composition of the film (Figs. 12, 13, 15), and the formation of tribofilms on carbon steel (Fig. 3), stainless steel and ceramic surfaces (Fig. 17).

The durability of the films (Fig. 16) is unexpected for physisorption, particularly at elevated temperatures. However, it is an important and interesting property for the application at hand. The casing connections need to maintain seal ability at elevated temperatures under micro sliding conditions. The presence and generation of a durable tribofilm can be beneficial for their performance. However, attention should be paid to the phosphate coating thickness. If the coating is not acting as a shear layer, tribofilm formation is limited or non-existent.

## Conclusions

The formation of tribofilms in a phosphate conversion coated tribosystem was investigated for the first time. Phosphate-based tribofilms derived from the hureaulite crystals on the disc were created on the ball counter surface in a pin-on-disc tribometer under variation of normal force, sliding length, sliding velocity, bulk temperature and base oil polarity.

The results indicate the following:

1. When rubbing against a manganese phosphate (hureaulite) coating under lubricated conditions an amorphous and durable tribofilm that consists of hureaulite is created.
2. The hureaulite particles are generated by cleavage along the {100}-plane which exposes phosphate groups. These are subsequently activated by shear stress which deforms or stretches the P–O bond(s). This leads to a more polar functional group which adsorbs at the ball surface forming a tribofilm.
3. Therefore, the formation of the tribofilm is activated by shear stress and the rate of formation of the tribofilms is strongly correlated with the magnitude of the shear stress in the tribological contact.
4. The initiation of the tribofilm is weakly correlated with the base oil polarity.
5. Tribofilm growth is only observed in in situ.
6. The adsorption mechanism is physisorption, confirmed by the tribofilm formation on carbon steel, stainless steel, silicon nitride and aluminium oxide, the unchanged chemical composition/structure and the absence of thermal acceleration.
7. No tribofilm formation was observed on thick ($>3\,{\text {g }}{\text {cm}}^{-2}$) phosphate coatings.

The conclusions fortify those in our earlier work [3]. Phosphate conversion coatings are a fact of life in the oil and gas industry and can be used as engineering parameters to develop the next generation environmentally friendly lubricants and ensure seal ability of casing connections.

## References

[1] Bommer P (2008). A Primer of Oilwell Drilling.

[2] Ernens D, de Rooij M, Pasaribu H, van Riet E, van Haaften WM, Schipper D (2018). Mechanical characterization and single asperity scratch behaviour of dry zinc and manganese phosphate coatings. Tribol. Int..

[3] Ernens, D., van Riet, E.J., de Rooij, M.B., Pasaribu, H.R., van Haaften, W.W.M., Schipper, D.J.: The role of phosphate conversion coatings in make-up and seal ability of casing connections. SPE Drill. Complet. (2018). 10.2118/184690-PA

[4] Ertas A (1992). Experimental investigation of galling resistance in OCTG connections. J. Manuf. Sci. Eng..

[5] Inose K, Sugino M, Goto K (2016). Influence of grease on high-pressure gas tightness by metal-to-metal seals of premium threaded connections. Tribol. Online.

[6] Rausch W (1990). The Phosphating of Metals.

[7] Nevosad A, Azhaarudeen S, Doerr N, Zacharias H, Klarner J, Badisch E (2016). Initial damage mechanism and running-in behaviour of phosphate conversion coatings. Key Eng. Mater..

[8] Perry J, Eyre T (1977). The effect of phosphating on the friction and wear properties of grey cast iron. Wear.

[9] Hivart P, Hauw B, Bricout J, Oudin J (1997). Seizure behaviour of manganese phosphate coatings according to the process conditions. Tribol. Int..

[10] Neville A, Morina A, Haque T, Voong M (2007). Compatibility between tribological surfaces and lubricant additives How friction and wear reduction can be controlled by surface/lube synergies. Tribol. Int..

[11] Azhaarudeen S, Faruck AAM, Nevosad A (2018). Tribological behaviour and wear mechanisms of manganese phosphate coatings under dry reciprocating sliding contact conditions. Tribol. Int..

[12] Le HR, Stewart F, Williams JA (2015). A simplified model of surface burnishing and friction in repeated make-up process of premium tubular connections. Tribol. Lett..

[13] Pérez-Ràfols F, Larsson R, Almqvist A (2016). Modelling of leakage on metal-to-metal seals. Tribol. Int..

[14] Pérez-Ràfols F, Larsson R, Lundström S, Wall P, Almqvist A (2016). A stochastic two-scale model for pressure-driven flow between rough surfaces. Proc. R. Soc. A.

[15] Pérez-Ràfols F, Larsson R, van Riet EJ, Almqvist A (2018). On the flow through plastically deformed surfaces under unloading: a spectral approach. Proc. Inst. Mech. Eng. C.

[16] Hill R, Jones J (1976). The crystal structure of hopeite. Am. Mineral..

[17] Moore PB, Araki T (1973). Hureaulite: lts atomic arrangement. Am. Mineral..

[18] Narayanan TSNS (2005). Surface pretreatment by phosphate conversion coatings—a review. Rev. Adv. Mater. Sci..

[19] Bosman R, Schipper DJ (2011). Mild wear prediction of boundary-lubricated contacts. Tribol. Lett..

[20] Stachowiak G, Batchelor AW (2013). Engineering Tribology.

[21] Beyer MK, Clausen-Schaumann H (2005). Mechanochemistry: the mechanical activation of covalent bonds. Chem. Rev..

[22] Spikes H (2015). Friction modifier additives. Tribol. Lett..

[23] Lansdown AR (2003). Lubrication and Lubricant Selection: A Practical Guide.

[24] Hsu S (1997). Boundary lubrication: current understanding. Tribol. Lett..

[25] Erdemir A (2005). Review of engineered tribological interfaces for improved boundary lubrication. Tribol. Int..

[26] Crobu M, Rossi A, Mangolini F, Spencer ND (2010). Tribochemistry of bulk zinc metaphosphate glasses. Tribol. Lett..

[27] Barnes AM, Bartle KD, Thibon VR (2001). A review of zinc dialkyldithiophosphates (ZDDPS): characterisation and role in the lubricating oil. Tribol. Int..

[28] Spikes H (2004). The history and mechanisms of ZDDP. Tribol. Lett..

[29] Gosvami NN, Bares JA, Mangolini F, Konicek AR, Yablon DG, Carpick RW (2015). Mechanisms of antiwear tribofilm growth revealed in situ by single-asperity sliding contacts. Science.

[30] Naveira Suarez A, Grahn M, Pasaribu R, Larsson R (2010). The influence of base oil polarity on the tribological performance of zinc dialkyl dithiophospate additives. Tribol. Int..

[31] Fujita H, Spikes HA (2004). The formation of zinc dithiophosphate antiwear films. Proc. Inst. Mech. Eng. Part J.

[32] Zhmud B (2007). Beyond the aniline point: critical solution point for the oil/aniline system as a measure of oil solubility. Fuel.

[33] Bosman R, de Rooij MB (2010). Transient thermal effects and heat partition in sliding contacts. J. Tribol..

[34] Kasap SO (2013). Optoelectronics & Photonics:Principles & Practices: International Edition.

[35] Cullity B, Stock S (2001). Element of X-ray Diffraction.

[36] Smith GC (2005). Evaluation of a simple correction for the hydrocarbon contamination layer in quantitative surface analysis by XPS. J. Electron Spectrosc. Relat. Phenom..

[37] Holmberg K, Matthews A (2009). Coatings Tribology: Properties, Mechanisms, Techniques and Applications in Surface Engineering.

[38] Shibata J, Fujii K, Murayama N, Yamamoto H (2002). Dispersion and flocculation behavior of fine metal oxide particles in various solvents. KONA Powder Part. J.

[39] Fujita H, Spikes HA (2005). Study of zinc dialkyldithiophosphate antiwear film formation and removal processes. Part II: kinetic model. Tribol. Trans..

[40] Zhang J, Spikes H (2016). On the mechanism of ZDDP antiwear film formation. Tribol. Lett..

[41] Ashcroft NW, Mermin ND (1976). Solid State Physics.

